# A Comprehensive Comparative Analysis of Deep Learning Based Feature Representations for Molecular Taste Prediction

**DOI:** 10.3390/foods12183386

**Published:** 2023-09-09

**Authors:** Yu Song, Sihao Chang, Jing Tian, Weihua Pan, Lu Feng, Hongchao Ji

**Affiliations:** 1 Zhengzhou Research Base, State Key Laboratory of Cotton Biology, School of Agricultural Sciences, Zhengzhou University, Zhengzhou 450001, China; syusalutezdys@gmail.com; 2 Shenzhen Branch, Guangdong Laboratory for Lingnan Modern Agriculture, Shenzhen 518120, China; 3 Genome Analysis Laboratory of the Ministry of Agriculture and Rural Affairs, Agricultural Genomics Institute at Shenzhen, Chinese Academy of Agricultural Sciences, Shenzhen 518120, China

**Keywords:** molecular feature representation, cheminformatics, taste prediction, machine learning, deep learning

## Abstract

Taste determination in small molecules is critical in food chemistry but traditional experimental methods can be time-consuming. Consequently, computational techniques have emerged as valuable tools for this task. In this study, we explore taste prediction using various molecular feature representations and assess the performance of different machine learning algorithms on a dataset comprising 2601 molecules. The results reveal that GNN-based models outperform other approaches in taste prediction. Moreover, consensus models that combine diverse molecular representations demonstrate improved performance. Among these, the molecular fingerprints + GNN consensus model emerges as the top performer, highlighting the complementary strengths of GNNs and molecular fingerprints. These findings have significant implications for food chemistry research and related fields. By leveraging these computational approaches, taste prediction can be expedited, leading to advancements in understanding the relationship between molecular structure and taste perception in various food components and related compounds.

## 1. Introduction

The sense of taste plays a pivotal role in determining our preferences and responses to various food components, and it is associated with specific organisms and survival needs [1]. For instance, the bitter taste acts as a protective mechanism against potentially toxic substances, although not all bitter compounds are inherently harmful. Intriguingly, research has revealed the presence of bitter ingredients in diverse sources such as clinical drugs, fruits, and vegetables [2]. On the other hand, sweeteners have the ability to enhance the perception of sweetness by interacting with specific receptors. However, excessive consumption of sweeteners can have adverse health effects, including the development of type-2 diabetes, heart disease, and other obesity-related conditions [3].

Taste prediction, a vital area of molecular study within food chemistry, encompasses the analysis and understanding of fundamental senses such as sweetness, bitterness, umami, acidity, and saltiness. It plays a crucial role in identifying and analyzing various factors, including condiments, sweet substitutes, and the underlying causes of bitterness in food [4].

Machine learning algorithms can be trained on existing datasets of molecular structures and associated taste properties to uncover intricate patterns and relationships. One of the key factors that significantly influences the accuracy and reliability of results is the molecular representation. Most commonly used are sets of physicochemical properties and various fingerprinting methods, which are applied in various previous studies [5,6,7,8,9,10,11,12].

For example, Cristian Rojas et al. proposed a Quantitative Structure–Taste Relationship (QSTR) expert system to predict the sweetness of molecules based on conformation-independent extended-connectivity fingerprints (ECFPs) and molecular descriptors. This QSTR model can better understand the relationship between molecular structure and sweetness, which can be used for the design of new sweeteners [9]. Suqing Zheng’s team trained machine learning models named e-bitter and e-sweet based on ECFPs and 2-D Dragon descriptors to predict sweeteners and bitterants, respectively [8,10]. Weichen Bo et al. established a regression-based model that can predict the structure–taste relationship. They used trained deep learning algorithms based on neural networks based on RDKit Fingerprints, molecular descriptors, and molecular images [12]. Tuwani et al. introduced a machine learning technique for predicting both the bitterness and sweetness of small molecules. Their methodology involved the application of the Boruta algorithm for feature selection and principal component analysis for dimensionality reduction. Classification models were then constructed using algorithms like random forest (RF), ridge logistic regression, and AdaBoost. To decipher the critical features and feature groups influencing bitter and sweet perceptions, SHAP analysis was employed [11]. Goel et al. explored the use of diverse regression models, such as RF, MLP, GBR, XGB, etc., to predict molecular sweetness. Through a comprehensive evaluation of these regression models, it was determined that GBR and RF yielded the most accurate results in sweet taste prediction, boasting correlation coefficients of 0.94 and 0.92, respectively [6]. Dagan-Wiener et al. devised a tool named BitterPredict (1.0), aimed at forecasting the bitterness of molecules through an analysis of their chemical structures. By utilizing the QikProp (3.9) software, they computed various physicochemical and ADME/Tox properties. Subsequently, an AdaBoost classifier was constructed [13].

Recently, advancements in deep learning have further enhanced the power and flexibility of molecular representations. Convolutional neural networks (CNNs), recurrent neural networks (RNNs), and graph neural networks (GNNs) have been extensively utilized in diverse applications like drug target interaction prediction [14,15,16,17,18], molecular property prediction [19,20,21,22,23], and genetic biology [24,25]. These methods utilize SMILES (Simplified Molecular Input Line Entry System), a textual representation for chemical structures, as input. This minimizes information loss compared to molecular fingerprints. SMILES encodes molecules through ASCII characters, representing atoms, bonds, and connectivity, akin to natural language. This encoding can be transformed into binary vectors via one-hot encoding, facilitating machine-readable data (CNN and RNN). Furthermore, SMILES can be converted into unbiased geometric topology data for machine processing (GNN). Recently, Shampa Raghunathan conducted an assessment of deep-learning based molecular representation methods, focusing on their underlying principles, implementation, and practical applications [26]. However, the comparison of performance of deep learning in taste prediction is lacking. Given the transformative impact of deep learning across multiple domains, there is a promising potential for its application in advancing the realm of in silico taste prediction. This data-centric methodology possesses the capability of complementing empirical assays and yielding invaluable insights into the molecular factors dictating various taste perceptions.

In this manuscript, we present a comprehensive investigation into the performance of established and recently proposed molecular feature representations in the context of taste prediction. The research primarily focuses on evaluating the efficacy of various molecular feature representations, with the aim of pinpointing the most suitable neural network architecture that can provide improved accuracy and versatility in the context of datasets centered around small molecules. Through this endeavor, we hope to provide valuable insights into the extent to which these representations encapsulate taste-related details and their suitability for different taste prediction tasks. 

## 2. Materials and Methods

### 2.1. Data Preparation

The dataset used in this study is sourced from ChemTastesDB [4], an extensive database comprising 2944 organic and inorganic tastants. The dataset includes essential information such as the name, PubChem CID, CAS registry number, canonical SMILES, taste category, and reference literature, providing a comprehensive foundation for our research. These tastants are classified into nine categories, encompassing five basic taste types (sweet, bitter, sour, umami, and salty) and four additional categories (tasteless, non-sweet, multi-taste, and miscellaneous). Specifically, the dataset consists of 977 sweet molecules, 1183 bitter molecules, 98 umami molecules, 38 sour molecules, 12 salty molecules, 113 multi-taste molecules, 203 tasteless molecules, 233 non-sweet molecules, and 87 miscellaneous molecules.

**Figure 1:**
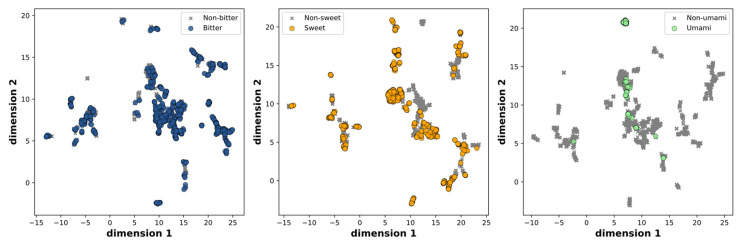
Scatter plot of the UMAP dimensions. Molecules are colored on the basis of bitter, sweet, and umami.

To ensure data quality and avoid redundancies, we initially excluded the multi-taste and miscellaneous molecules, resulting in a dataset of 2744 molecules. Subsequently, we removed duplicate entries, resulting in a final dataset of 2601 molecules. These molecules were further classified into three categories based on their taste characteristics: sweet and non-sweet, bitter and non-bitter, and fresh and non-fresh. The distribution across these categories is as follows: 906 sweet and 1695 non-sweet molecules, 1126 bitter and 1475 non-bitter molecules, and 98 umami and 2503 non-umami molecules, respectively. The chemical space was visualized by evaluating molecular similarity and diversity using UMAP, which compressed the 166-dimensional binary vectors in the MACCS keys into a 2D representation. This mapping effectively portrays the distribution of positive and negative samples in Figure 1, both of which are uniformly spread within the chemical space.

To conduct our analysis, we randomly split the dataset into a training set, validation set, and test set, following a ratio of 7:1:2, ensuring that the distribution of molecules across the different taste categories remained representative in each subset. The detailed information can be found in Table 1.

### 2.2. Molecular Representation

**Figure 2:**
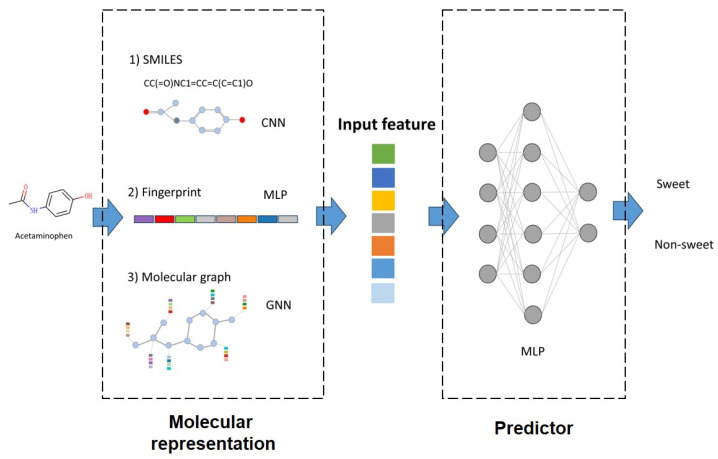
Visual representation of input, molecular embedding, and classifier of the utilized models.

Fingerprints, convolutional neural networks (CNN), and graph neural networks (GNN) are most widely used molecular representation strategies in Quantitative Structure–Activity Relationship (QSAR) studies [27]. These methods have indeed demonstrated their effectiveness in various molecular modeling tasks. In this study, we assess these methods and evaluate their applicability to the taste prediction tasks. The implementation is assisted by the DeepPurpose (0.1.5) package, which is a molecular modeling and prediction toolkit integrating numerous molecular representation methods [18]. The inputs, outputs, and model interpretation are summarized in Figure 2. 

#### 2.2.1. Fingerprint

Molecular fingerprints encode structural patterns of molecules into binary vectors as the input of the prior predictor. Six distinct molecular fingerprints or descriptors were used for comparison with deep-learning based representation. These molecular representations capture different aspects of chemical structures, which are briefly described as follows:

(1) Morgan fingerprint [28]: A circular fingerprint encoding structural information by considering substructures at different radii around each atom.
(2) PubChem fingerprint [29]: A binary fingerprint derived from the PubChem Compound database, representing molecular structural features based on predefined chemical substructures.
(3) Daylight fingerprint: A descriptor developed by Daylight Chemical Information Systems, encoding chemical features by identifying fragments and substructures within a molecule.
(4) RDKit fingerprint: A fingerprinting method integrated by the RDKit package. It is a dictionary with one entry per bit set in the fingerprint; the keys are the bit IDs; the values are tuples of tuples containing bond indices.
(5) ESPF fingerprint [18]: An explainable substructure partition fingerprint capturing extended connectivity patterns within a molecule, representing the presence of specific atom types and their surrounding environments.
(6) ErG fingerprint [30]: A novel fingerprinting method presented that uses pharmacophore-type node descriptions to encode the relevant molecular properties.

#### 2.2.2. Convolutional Neural Network 

Convolutional neural network (CNN) based molecular embedding takes molecular SMILES strings as input. These strings are treated similarly to natural language, being converted into one-hot encoding, after which convolutional layers are utilized to generate numerical representations. Three kinds of models were used for comparison, which are briefly described as follows:

(1) Simple CNN [31]:

The CNN model takes the Simplified Molecular Input Line Entry System (SMILES), a notation system used to represent the structure of a molecule using ASCII characters, as input, which was previously used in drug-target prediction [32]. A one-hot strategy is used for transforming the strings into a two-dimensional array. Three one-way convolutional layers are followed by max pooling layers to extract meaningful features from the input SMILES string. The architecture includes ReLU activation functions to introduce non-linearity in the neural network so as to fit more complex data distributions. We have carefully chosen 32, 64, and 96 as the number of filters; 4, 6, and 8 as the kernel sizes; and 1 as the stride as hyperparameters to optimize performance.

(2) CNN-LSTM [33]:

The CNN_LSTM model incorporates LSTM layers following the CNN layers. The LSTM layer is a widely utilized recurrent neural network structure that effectively captures long sequence dependencies. In contrast to regular RNNs, LSTM employs forget gate, input gate, and output gate mechanisms, enabling selective retention and omission of input and historical information. Consequently, it excels in modeling lengthy sequences by preserving essential information. In this model, bidirectional LSTM layers are employed with the parameter bidirectional = True. Additionally, the model includes two LSTM units with num_layers = 2. By leveraging bidirectional LSTM, the model can encode contextual information and comprehend semantic dependencies within the input.

(3) CNN-GRU [33]:

The CNN_GRU model merges the CNN and GRU architectures. The CNN component utilizes one-dimensional convolution to extract relevant features from the sequence, while the GRU component captures long-term dependencies within the sequence. Specifically, the GRU consists of two hidden layers, each containing 64 hidden units. By adjusting the parameters, we configure it to be a bidirectional GRU. Compared to LSTM, GRU has a simpler structure, featuring only an update gate and a reset gate. The reset gate enables control over the retention of past states, while the update gate governs the extent to which the new state replicates the old state.

#### 2.2.3. Graph Neural Networks

In this study, we employ five distinct graph neural network (GNN) models integrated with Life [34] and DeepPurpose for comparative analysis. GNN models collectively treat molecules as graph data and extract information from the molecular structure using diverse methodologies. Here is a brief description of each approach:

(1) GCN [35]:

The GCN model utilizes graph convolutional neural networks (GCN) to extract features. Initially, the input SMILES strings are transformed into molecular graphs, which are graphical representations of molecules where atoms are represented as nodes and bonds between atoms are represented as edges. From these graphs, features are extracted with GCN. The model comprises three GCN layers, each consisting of 64 hidden units. To ensure stable training, residual connection layers and batch normalization layers are incorporated. Following the three GCN layers, aggregation layers are employed to consolidate node features into graph-level features. Lastly, fully connected layers are used to map the features into a 256-dimensional space.

(2) NeuralFP [36]:

NeuralFP is a variation of GCN that introduces multiple layers of message passing to capture higher-order neighbor information of nodes. It takes into account both “left neighbors” and “right neighbors” in two directions during the message passing process. Following each layer of the graph neural network, batch normalization layers are applied to accelerate model convergence and enhance its stability. Various parameter configurations were explored. To avoid excessive model complexity while considering left and right neighbors of nodes, we set the maximum degree to 10. The dimension of graph features is set to 128, influencing the representation of the graph. Additionally, the activation function tanh is employed to enhance the model’s non-linear capabilities. By appropriately setting these parameters, effective optimization of the model can be achieved.

(3) GIN-AttrMasking [37]:

The GIN-AttrMasking model utilizes graph isomorphic networks (GIN) as its underlying architecture. Initially, node features and edge features are embedded. Subsequently, five GIN layers are applied, with each layer comprising two fully connected layers activated by ReLU functions. Following this, 300-dimensional embeddings are assigned to different edge types. A normalization layer is then introduced and average pooling is performed on the nodes to obtain the graph’s overall representation. To mitigate overfitting, a dropout rate of 0.1 is applied between the fully connected layers.

(4) GIN-ContextPred [37]:

The GIN-ContextPred model is similar to the GIN-AttrMasking model. In the GIN layers, the central node’s representation is concatenated with the representations of its neighboring nodes. This incorporation of contextual information allows the model to capture a more comprehensive representation, enhancing its ability to learn node representations effectively.

(5) AttentiveFP [38]:

AttentiveFP is a graph neural network enhanced with an attention mechanism. It first obtains initial context representations of nodes and edges through the GetContext layer. Internally, it uses an Attentive GRU module that performs weighted summation of edge representations based on attention scores to update the context node representations. The subsequent GNNLayer is the basic layer for message passing and node representation updating. It also uses Attentive GRU internally. Finally, the AttentiveFP readout module, which contains two GlobalPool layers with LeakyReLU activation functions, extracts graph-level representations from the node representations.

### 2.3. Predictor

After feature representation, the molecules are embedded into vectors; a predictor is used for classification. Commonly used classifiers such as multilayer perceptron (MLP) random forest (RF), support vector machine (SVM) and naive Bayes are also compared, of which results are summarized in Tables S1–S3. Since MLP is almost ranked as top, and it can be seamlessly joined with CNN and GNN molecular embedders, MLP is taken as the unitive predictor in the following procedures.

In the MLP predictor, dropout layers are then incorporated into the model, randomly deactivating some neurons with a dropout rate of 0.1. This is done to prevent overfitting and enhance the model’s generalization capabilities. Following the dropout layer, a fully connected layer called the predictor is added, consisting of two linear layers. The first layer transforms the 256-dimensional features into 512 dimensions, while the second layer further transforms them into the output layer. The output consists of the probabilities indicating whether a molecule possesses the specific taste or not.

Binary cross-entropy is employed as the loss function to calculate the error between the predicted probability and the true label. The Adam optimization algorithm is utilized to optimize the model parameters which adjusts the learning rate based on the historical and current gradients of the parameters. During the training process, the classifier is initially constructed by encoding the molecules with an initial learning rate of 0.001 and optimized using the Adam optimizer. The training consists of 20 epochs, with each epoch updating the parameters using a batch size of 64 from the training set. The model’s performance is evaluated by monitoring indicators on the validation set to save the optimal model.

## 3. Results

### 3.1. Evaluation Metrics

The performance of the models was assessed using multiple evaluation metrics, including accuracy, precision, sensitivity, specificity, F1 score, area under the receiver operating characteristic curve (AUROC), and area under the precision–recall curve (AUPRC). Each metric provides valuable insights into different aspects of the model’s performance.

Precision measures the proportion of predicted positive samples that actually belong to the positive class. It quantifies the model’s ability to accurately identify positive compounds. Sensitivity represents the true positive rate, indicating the number of positive compounds correctly predicted as positive. Specificity indicates the number of negative compounds correctly predicted as negative. It evaluates the model’s ability to correctly identify negative compounds. F1 score combines precision and sensitivity, providing a balanced measure of the model’s performance. It is especially valuable for evaluating classification models and considering the trade-off between false positives and false negatives.

AUROC evaluates the overall discriminative power and balanced prediction performance of the model. Additionally, auxiliary indicators such as accuracy (ACC), AUPRC, and F1 score were employed. AUPRC, similarly to AUROC, serves as a balanced prediction evaluation metric, particularly in scenarios with highly imbalanced data. It is particularly effective in assessing models’ performance when dealing with imbalanced datasets.

The calculation formulas for the aforementioned metrics are as follows:

(1) $$Accuracy=\frac{TP+TN}{TP+FP+TN+FN}$$

(2) $$precision=\frac{TP}{TP+FP}$$

(3) $$Recall\;or\;Sensitivity=\frac{TP}{TP+FN}$$

(4) $$specificity=\frac{TN}{TN+FP}$$

(5) $$F1\;score=\frac{2\cdot Precision\cdot Recall}{Precision+Recall}$$

Here *TP* represents true positives, *TN* represents true negatives, *FP* represents false positives, and *FN* represents false negatives.

### 3.2. Comparison of Model Performance

**Figure 3:**
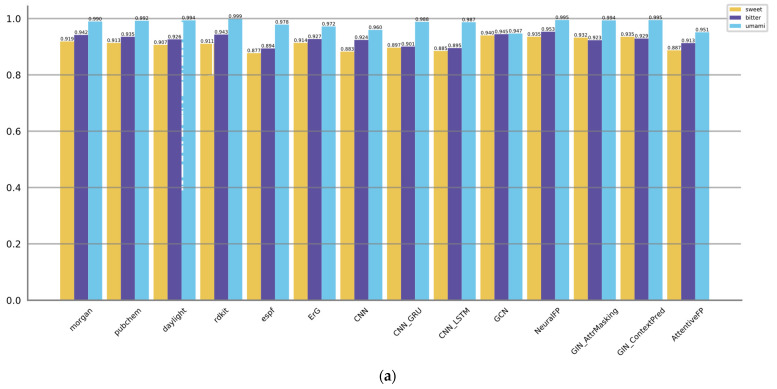

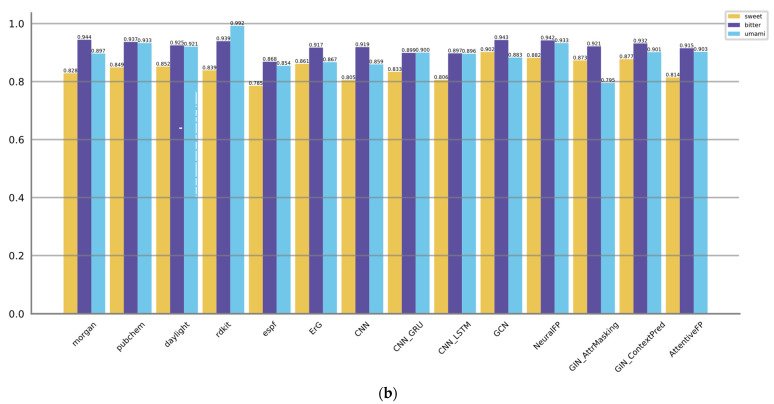
Showcases of the performance of different molecular representation models in predicting sweet, bitter, and umami tastes, as indicated by their AUROC scores (**a**) and AUPRC scores (**b**).

First, we evaluated the performance of the three types of 14 representation models on predicting the molecular tastes. The results are summarized in Table 2, Table 3 and Table 4. Figure 3 displays the AUROC and AUPRC values. Metrics of model complexity are summarized in Table S4. Metrics of training stages are provided in Tables S5–S7.

For predicting sweet taste, the GNN-based models (GCN, NeuralFP) and fingerprint-based models (Morgan, PubChem, ErG) exhibit better performance metrics compared to the CNN-based models. Notably, GCN and NeuralFP stand out by achieving high accuracies of 0.869 and 0.896, respectively. Moreover, these models showcase excellent F1 scores of 0.813 and 0.812, underscoring their efficacy in accurately predicting the sweetness of molecules. Furthermore, these models showcase satisfactory precision, sensitivity, and specificity, suggesting a balanced ability to correctly identify both positive and negative samples. It can also be proved by AUROC and AUPRC values; GCN and NeuralFP are also listed as the top two. 

When it comes to predicting bitter taste, both GNN-based models (GCN, NeuralFP) and fingerprint-based models (Morgan, PubChem, RDKit) consistently outperform the CNN-based models. However, it is important to note that the GNN-based models do not have a significant advantage over the fingerprint-based models in predicting bitter molecules. NeuralFP remains the top performer in the GNN category, achieving the highest accuracy of 0.896 and F1 score of 0.885. On the other hand, the fingerprint-based models, specifically Pubchem and RDKit, achieve comparable results. Pubchem achieves an accuracy of 0.879 and an F1 score of 0.865, while RDKit achieves an accuracy of 0.869 and an F1 score of 0.857. AUROC and AUPRC values of NeuralFP, GCN, Morgan, PubChem, and RDKit are also listed as the top five. These results suggest that these models achieve a strong precision–recall trade-off and perform well across various threshold levels. This implies that these models are effective in accurately predicting the target outcomes while achieving a balance between precision and recall.

Regarding the umami taste, the majority of methods exhibit satisfactory performance with accuracy levels generally surpassing 0.97, while the F1 scores typically exceed 0.70. This may be attributed to the relatively simpler nature of the prediction task compared to the aforementioned ones. Pubchem, AttentiveFP, GCN, Morgan, ErG, and CNN_GRU exhibit slightly superior performance compared to the other methods, as they achieve higher accuracies and F1 scores. The notably high precision and specificity values suggest that these models have a low rate of false positives, which could potentially be influenced by the presence of an imbalance in the training data. 

### 3.3. Voting/Consensus Model Performance

Following that, we proceed with a voting/consensus strategy to investigate the potential enhancement in taste prediction performance through consensus. In this approach, we utilize the average predicting probabilities obtained from multiple models as the final decision, referred to as the “Ensemble score”. Various types of consensus approaches are employed, including:

(1) Consensus FP: The ensemble score is obtained by voting from six molecular fingerprint methods.
(2) Consensus CNN: The ensemble score is obtained by voting from three CNN methods.
(3) Consensus GNN: The ensemble score is obtained by voting from five GNN methods.
(4) FP + CNN: This approach combines the top two molecular fingerprint methods and the top two CNN methods based on their best F1 scores.
(5) FP + GNN: This approach combines the top two molecular fingerprint methods and the top two GNN methods based on their best F1 scores.
(6) CNN + GNN: This approach combines the top two CNN methods and the top two GNN methods based on their best F1 scores.
(7) FP + CNN + GNN: This approach combines the top two molecular fingerprint methods, the top two CNN methods, and the top two GNN methods based on their best F1 scores.

**Figure 4:**
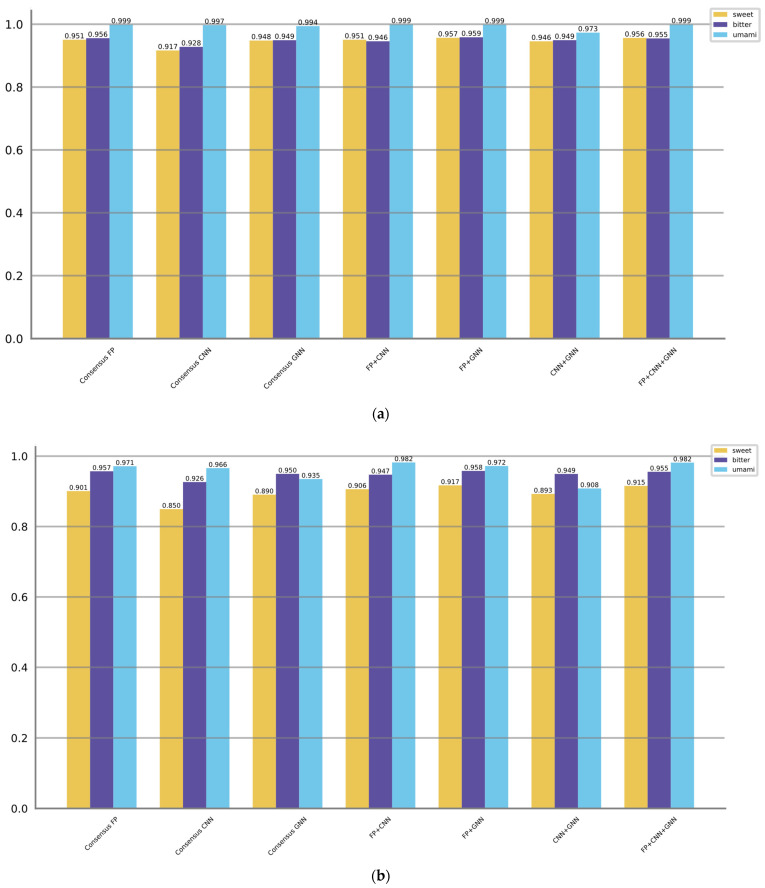
Showcases of the performance of the voting/consensus models in predicting sweet, bitter, and umami tastes, as indicated by their AUROC scores (**a**) and AUPRC scores (**b**).

In the above descriptions, “top x” refers to selecting the x models with the best F1 scores. The evaluation results of the ensemble approaches are presented in Table 5, Table 6 and Table 7. Additionally, Figure 4 displays the AUROC and AUPRC values corresponding to the different ensemble strategies. 

When predicting sweet taste, Consensus FP, Consensus CNN, and Consensus GNN demonstrate superior performance compared to their individual models within the fingerprint, CNN, and GNN categories, as indicated by higher F1 scores, AUROC values, and AUPRC values. The enhanced performance can be attributed to various factors inherent in the consensus strategy such as combining diverse information, mitigating individual model biases, robustly handling variability, and aggregating complementary information. Moreover, the consensus models that combine multiple categories (FP + GNN, FP + CNN + GNN) can exhibit superior performance compared to the consensus model within a single category. However, the CNN + GNN and FP + CNN combinations are not as good as Consensus GNN and Consensus FP, which takes into account the initially poor performance of the CNN-based models. Among all the models, the FP + GNN model demonstrates superior performance in predicting sweet taste with optimal F1, AUROC, and AUPRC scores of 0.852, 0.957, and 0.917, respectively. 

The same trend persists when predicting bitter taste, where the FP + GNN model achieves the highest F1 (0.882), AUROC (0.959), and AUPRC (0.958), followed by FP + CNN + GNN. However, when it comes to umami taste prediction, the performance among models is relatively comparable. Consensus models show either no improvement or only slight improvements.

Based on the aforementioned comparisons, we can deduce that incorporating global chemical information through molecular fingerprints, which capture molecular composition, along with topological information obtained from graph structures, enables more comprehensive feature learning. This comprehensive feature learning leads to improved performance in taste prediction tasks.

### 3.4. In Silico Compound Taste Database

As an application of molecular taste prediction, an in silico compound taste database is built based on the FP + GNN model, which performs best in the test tasks. In order to provide a comprehensive collection of molecular structures associated with various tastes, FooDB (https://foodb.ca/downloads) (accessed on 3 May 2023) is employed to access a vast array of compound structures. Within this website lies a rich repository of chemical information on food components, facilitating the exploration of the molecular basis of taste perception by researchers.

The prediction process encompassed the conversion of the molecular structures obtained from the database into appropriate input representations for the FP + GNN model. The predicted compounds with taste characteristics, namely, sweet, bitter, and umami, are collected. We believe the result will facilitate finding potential additives, determining consumer preferences and/or enhancing the flavor of food products. This in silico database is available in Tables S8–S10 of the Supplementary Materials.

## 4. Discussion

In this study, we conducted a comprehensive comparison of various molecular representation methods for three taste prediction tasks. The primary objective was to ascertain the applicability of deep learning-driven molecular representation techniques and to identify the optimal approaches for addressing taste prediction tasks, an aspect that has been notably absent in previous research endeavors. Since previous authors have summarized the performance of the taste prediction tools referred to in their original papers [39,40], we alternatively examined the performance of various methods with a consistent and comprehensive database. In addition, we employed a robust set of metrics to gauge their effectiveness. Our emphasis was primarily on deep learning based molecular representation methods, which is an aspect that had not been extensively addressed in earlier reviews. While Tomaz Stepisnik performed a comparison across seven prevalent QSAR tasks and indicated a lack of enhancement with graph convolutional networks (GCN) in contrast to molecular fingerprints [41], it is important to highlight that the suitability of distinct molecular representation methods could vary across different tasks. 

To summarize, umami taste prediction is relatively straightforward as most methods perform similarly well overall. However, when it comes to sweet and bitter prediction, the top-performing methods typically fall within the GNN category. This observation suggests that the graph structure built from atoms and bonds effectively captures the key molecular characteristics associated with taste. Furthermore, the success of GNN-based models in taste prediction implies that specific molecular features encoded in the graph structure, such as functional groups, aromatic systems, or spatial arrangements, have a strong influence on the perception of sweetness and bitterness. GNN based techniques have demonstrated success in numerous prediction tasks, particularly those that exhibit strong associations with functional groups, such as chromatographic retention time [23], synthetic accessibility [42], and compound protein interaction [43]. In particular, taste prediction aligns with compound–protein interactions at its core, as tastes are mediated by distinct receptors [44]. Consequently, we hold the viewpoint that GNN methods are poised for promising outcomes in taste prediction, especially when the dataset size matches that of drug target data.

The consensus model FP + GNN outperforms other consensus models which indicates the complementary strengths of the two different representation approaches: GNN and molecular fingerprints. The GNN models excel in capturing the inherent spatial and connectivity information of molecules by considering the relationships between atoms and their neighbors in the molecular graph. This allows them to learn and represent complex structural patterns that are crucial for taste prediction. On the other hand, molecular fingerprints provide a concise representation of the overall molecular composition, encoding key structural features and substructures. They are efficient in capturing global molecular characteristics and can be useful in encoding higher-level properties related to taste perception. The hypothesis can be supported by the points that their fusion can also be employed in alternative manners. For instance, FP-GNN combines molecular embedding by merging fingerprint vectors and feature vectors extracted by GNN in a single neural network, resulting in improved property prediction performance [22].

By combining the strengths of GNN and molecular fingerprints through the consensus model, the predictive model can leverage both the fine-grained structural details learned by GNN and the overall molecular features captured by fingerprints. GNNs excel at capturing fine-grained structural details of molecules by recursively aggregating information from neighboring atoms and bonds. This makes GNNs particularly effective at learning local patterns, spatial relationships, and molecular interactions that contribute to the overall behavior of the molecule. Molecular fingerprints are compact binary or numerical representations of molecules that encode information about their chemical structure. They capture global molecular features, such as presence/absence of specific substructures or chemical properties. Molecular fingerprints are efficient for representing overall molecular characteristics. By combining both representations, the consensus model can capture both fine-grained structural information and global molecular features, leading to a more comprehensive representation of the molecule. This hybrid approach enables a more comprehensive understanding of molecular attributes related to taste, resulting in improved predictive performance. The utilization of this voting/consensus methodology presents a source of inspiration for other predictive modeling tasks in the realm of the Quantitative Structure–Activity Relationship (QSAR). In scenarios where diverse molecular facets contribute in a synergistic manner, this approach has the potential to enhance model performance and facilitate well-informed decision-making. This is particularly suggested when a solitary model fails to yield a satisfactory prediction; a similar strategy has also been applied in disease-related mutations [45].

Ultimately, we hope our study will not only contribute to the understanding of taste prediction within the realm of molecular representation but will also offer valuable insights into the broader landscape of predictive modeling in the field of food science.

## Figures and Tables

**Table 1:** The sweet, bitter, and umami datasets used in this study.

Category	Training		Validation		Test		Total
	Number	Positive Rate	Number	Positive Rate	Number	Positive Rate	
Sweet	637	0.350	91	0.350	178	0.342	906
Nonsweet	1184		169		342		1695
Bitter	769	0.422	118	0.454	239	0.460	1126
Non-bitter	1052		142		281		1475
Umami	71	0.039	8	0.031	19	0.037	98
Non-umami	1750		252		501		2503

**Table 2:** Performance comparison of 14 models for predicting sweet taste.

Model	TP	FP	TN	FN	Acc.	Prec.	Sens.	Spec.	F1
Morgan	151	57	285	27	0.838	0.726	0.848	0.833	0.782
Pubchem	152	61	281	26	0.833	0.714	0.854	0.822	0.777
Daylight	120	33	309	58	0.825	0.784	0.674	0.904	0.725
RDKit	127	35	307	51	0.835	0.784	0.713	0.898	0.747
ESPF	123	49	293	55	0.800	0.715	0.691	0.857	0.703
ErG	140	45	297	38	0.840	0.757	0.787	0.868	0.771
CNN	141	60	282	37	0.813	0.701	0.792	0.825	0.744
CNN_GRU	134	43	299	44	0.833	0.757	0.753	0.874	0.755
CNN_LSTM	114	29	313	64	0.821	0.797	0.640	0.915	0.710
GCN	148	38	304	30	0.869	0.796	0.831	0.889	0.813
NeuralFP	147	37	305	31	0.869	0.799	0.826	0.892	0.812
GIN_AttrMasking	154	52	290	24	0.854	0.748	0.865	0.848	0.802
GIN_ContextPred	152	52	290	26	0.850	0.745	0.854	0.848	0.796
AttentiveFP	98	19	323	80	0.810	0.838	0.550	0.944	0.664

**Table 3:** Performance comparison of 14 models for predicting bitter taste.

Model	TP	FP	TN	FN	Acc.	Prec.	Sens.	Spec.	F1
Morgan	197	30	251	42	0.862	0.868	0.824	0.893	0.845
Pubchem	202	26	255	37	0.879	0.886	0.845	0.907	0.865
Daylight	197	43	238	42	0.837	0.821	0.824	0.847	0.823
RDKit	203	32	249	36	0.869	0.864	0.849	0.886	0.857
ESPF	196	53	228	43	0.815	0.787	0.820	0.811	0.803
ErG	190	25	256	49	0.858	0.884	0.795	0.911	0.837
CNN	163	16	265	76	0.823	0.911	0.682	0.943	0.780
CNN_GRU	167	19	262	72	0.825	0.898	0.699	0.932	0.786
CNN_LSTM	173	25	256	66	0.825	0.874	0.724	0.911	0.792
GCN	193	27	254	46	0.860	0.877	0.808	0.904	0.841
NeuralFP	207	22	259	32	0.896	0.904	0.866	0.922	0.885
GIN_AttrMasking	174	23	258	65	0.831	0.883	0.728	0.918	0.798
GIN_ContextPred	169	14	267	70	0.838	0.923	0.707	0.950	0.801
AttentiveFP	170	19	262	69	0.831	0.899	0.711	0.932	0.794

**Table 4:** Performance comparison of 14 models for predicting umami taste.

Model	TP	FP	TN	FN	Acc.	Prec.	Sens.	Spec.	F1
Morgan	15	0	501	4	0.992	1.000	0.789	1.000	0.882
Pubchem	17	1	500	2	0.994	0.944	0.895	0.998	0.919
Daylight	16	5	496	3	0.985	0.762	0.842	0.990	0.800
RDKit	19	14	487	0	0.973	0.576	1.000	0.972	0.731
ESPF	15	4	497	4	0.985	0.789	0.789	0.992	0.789
ErG	16	1	500	3	0.992	0.941	0.842	0.998	0.889
CNN	13	3	498	6	0.983	0.813	0.684	0.994	0.743
CNN_GRU	15	0	501	4	0.992	1.000	0.789	1.000	0.882
CNN_LSTM	14	1	500	5	0.988	0.933	0.737	0.998	0.824
GCN	15	0	501	4	0.992	1.000	0.789	1.000	0.882
NeuralFP	17	7	494	2	0.982	0.708	0.895	0.986	0.791
GIN_AttrMasking	16	10	491	3	0.975	0.615	0.842	0.980	0.711
GIN_ContextPred	16	6	495	3	0.983	0.727	0.842	0.988	0.780
AttentiveFP	19	0	501	3	0.994	1.000	0.842	1.000	0.914

**Table 5:** Performance comparison of 7 consensus models for predicting sweet taste.

Model	TP	FP	TN	FN	Acc.	Prec.	Sens.	Spec.	F1
Consensus FP	141	24	318	37	0.883	0.855	0.792	0.930	0.822
Consensus CNN	138	41	301	40	0.844	0.771	0.775	0.880	0.773
Consensus GNN	142	23	319	36	0.887	0.861	0.798	0.933	0.828
FP + CNN	156	40	302	22	0.881	0.796	0.876	0.883	0.834
FP + GNN	153	28	314	25	0.898	0.845	0.860	0.918	0.852
CNN + GNN	141	26	316	37	0.879	0.844	0.792	0.924	0.817
FP + CNN + GNN	153	29	313	25	0.896	0.841	0.860	0.915	0.850

**Table 6:** Performance comparison of 7 consensus models for predicting bitter taste.

Model	TP	FP	TN	FN	Acc.	Prec.	Sens.	Spec.	F1
Consensus FP	205	27	254	34	0.883	0.884	0.858	0.904	0.870
Consensus CNN	181	19	262	58	0.852	0.905	0.757	0.932	0.825
Consensus GNN	188	13	268	51	0.877	0.935	0.787	0.954	0.855
FP + CNN	192	16	265	47	0.879	0.923	0.805	0.943	0.859
FP + GNN	202	17	264	37	0.896	0.922	0.845	0.940	0.882
CNN + GNN	189	13	268	50	0.879	0.956	0.791	0.954	0.857
FP + CNN + GNN	197	15	266	42	0.890	0.929	0.824	0.947	0.874

**Table 7:** Performance comparison of 7 consensus models for predicting umami taste.

Model	TP	FP	TN	FN	Acc.	Prec.	Sens.	Spec.	F1
Consensus FP	16	1	500	3	0.992	0.941	0.842	0.998	0.889
Consensus CNN	16	0	501	3	0.994	1.000	0.842	1.000	0.914
Consensus GNN	17	1	500	2	0.994	0.944	0.895	0.998	0.919
FP + CNN	15	0	501	4	0.992	1.000	0.789	1.000	0.882
FP + GNN	15	0	501	4	0.992	1.000	0.789	1.000	0.882
CNN + GNN	16	0	501	3	0.994	1.000	0.842	1.000	0.914
FP + CNN + GNN	15	0	501	4	0.992	1.000	0.789	1.000	0.882

## Data Availability

The code and testing data can be freely downloaded at https://github.com/songyu2022/taste_predict.git (accessed on 3 May 2023).

## Supplementary Materials

The following supporting information can be downloaded at: https://www.mdpi.com/article/10.3390/foods12183386/s1, Tables S1–S3: Performance metrics of different classifiers with Pubchem fingerprint for sweet, bitter, and umami taste; Table S4: Complexity of the utilized models; Tables S5–S7: Performance on training set of 14 models for sweet, bitter, and umami taste; Tables S8–S10: In silico molecular database of sweet, bitter, and umami taste.
