# Supplementary material for: A Comprehensive Comparative Analysis of Deep Learning Based Feature Representations for Molecular Taste Prediction
Source: Foods. 2023 Sep 9;12(18):3386. doi: 10.3390/foods12183386 (PMC10529232; doi:10.3390/foods12183386)
Supplement: Supplementary file 1 [file foods-12-03386-s001.zip › Table S1-S7.pdf]

Table S1. Performance metrics of different classifier with Pubchem fingerprint for sweet taste.

Model	Accuracy	Precision	Sensitivity	Specificity	F1 score
MLP+Pubchem	0.833	0.714	0.854	0.822	0.777
SVM+Pubchem	0.854	0.815	0.742	0.912	0.776
RF+Pubchem	0.867	0.819	0.787	0.909	0.802
Naive Bayes+Pubchem	0.712	0.563	0.708	0.713	0.627

Table S2. Performance metrics of different classifier with Pubchem fingerprint for bitter taste.

Model	Accuracy	Precision	Sensitivity	Specificity	F1 score
MLP+Pubchem	0.879	0.886	0.845	0.907	0.865
SVM+Pubchem	0.888	0.879	0.879	0.897	0.879
RF+Pubchem	0.890	0.899	0.858	0.918	0.878
Naive Bayes+Pubchem	0.7	0.686	0.640	0.751	0.662

Table S3. Performance metrics of different classifier with Pubchem fingerprint for umami taste.

Model	Accuracy	Precision	Sensitivity	Specificity	F1 score
MLP+Pubchem	0.994	0.944	0.895	0.998	0.919
SVM+Pubchem	0.994	1	0.842	1	0.914
RF+Pubchem	0.994	1	0.842	1	0.914
Naive Bayes+Pubchem	0.910	0.267	0.842	0.912	0.405

Table S4. Complexity of the utilized models.

Model	Number of modules	The number of parameters
Morgan	11	1477185
Pubchem	11	1330753
Daylight	11	2525761
Rdkit	11	633409
ESPF	11	3076673
ErG	11	751169
CNN	11	226625
CNN_GRU	12	3124033
CNN_LSTM	12	3169601
GCN	29	191810
NeuralFP	85	480193
GIN_AttrMasking	59	2067053
GIN_ContextPred	59	2067053
AttentiveFP	63	300806

Table S5. Performance on training set of 14 models for sweet taste.

Model	Accuracy	Precision	Sensitivity	Specificity	F1 score
Morgan	0.998	0.995	1	0.997	0.998
Pubchem	0.940	0.936	0.889	0.967	0.911
Daylight	0.968	0.980	0.928	0.990	0.953
RDKit	0.956	0.988	0.886	0.994	0.934
ESPF	0.997	0.997	0.995	0.998	0.996

ErG	0.907	0.849	0.893	0.915	0.871
CNN	0.954	0.899	0.980	0.941	0.938
CNN_GRU	0.871	0.881	0.730	0.947	0.798
CNN_LSTM	0.842	0.814	0.710	0.913	0.758
GCN	0.914	0.881	0.871	0.937	0.876
NeuralFP	0.897	0.957	0.739	0.982	0.834
GIN_AttrMasking	0.856	0.771	0.837	0.867	0.803
GIN_ContextPred	0.826	0.723	0.816	0.832	0.767
AttentiveFP	0.802	0.767	0.622	0.899	0.687

Table S6. Performance on training set of 14 models for bitter taste.

Model	Accuracy	Precision	Sensitivity	Specificity	F1 score
Morgan	0.997	0.995	0.999	0.996	0.997
Pubchem	0.960	0.987	0.917	0.991	0.951
Daylight	0.982	0.972	0.986	0.979	0.979
RDKit	0.976	0.984	0.959	0.988	0.972
ESPF	0.999	1	0.997	1	0.999
ErG	0.946	0.965	0.907	0.975	0.935
CNN	0.987	0.986	0.985	0.989	0.985
CNN_GRU	0.928	0.897	0.939	0.919	0.917
CNN_LSTM	0.884	0.948	0.766	0.970	0.847
GCN	0.951	0.940	0.944	0.956	0.942
NeuralFP	0.947	0.991	0.882	0.994	0.933
GIN_AttrMasking	0.901	0.970	0.789	0.982	0.870
GIN_ContextPred	0.901	0.976	0.785	0.986	0.870
AttentiveFP	0.852	0.877	0.757	0.922	0.812

Table S7. Performance on training set of 14 models for umami taste.

Model	Accuracy	Precision	Sensitivity	Specificity	F1 score
Morgan	1	1	1	1	1
Pubchem	1	1	1	1	1
Daylight	0.999	0.986	1	0.999	0.993
RDKit	1	1	1	1	1
ESPF	1	1	1	1	1
ErG	0.999	1	0.972	1	0.986
CNN	0.993	1	0.817	1	0.899
CNN_GRU	0.983	0.744	0.859	0.988	0.797
CNN_LSTM	0.990	1	0.732	1	0.846
GCN	0.997	1	0.930	1	0.964
NeuralFP	0.999	0.973	1	0.999	0.986
GIN_AttrMasking	0.993	0.845	1	0.993	0.916
GIN_ContextPred	0.992	0.843	0.986	0.993	0.909
AttentiveFP	0.987	0.885	0.761	0.996	0.818
